# Palforzia for Peanut Allergy: A Narrative Review and Update on a Novel Immunotherapy

**DOI:** 10.7759/cureus.50485

**Published:** 2023-12-13

**Authors:** Grant E Borne, Charles P Daniel, Maxwell J Wagner, Connor J Plaisance, Alexandra Nolen, Rucha A Kelkar, Shahab Ahmadzadeh, Dariusz Myrcik, Sahar Shekoohi, Alan D Kaye, Giustino Varrassi

**Affiliations:** 1 School of Medicine, Louisiana State University Health Sciences Center, Shreveport, USA; 2 School of Medicine, Medical University of South Carolina, Charleston, USA; 3 Department of Anesthesiology, Louisiana State University Health Sciences Center, Shreveport, USA; 4 Department of Internal Medicine, Emergency Medicine, Medical University of Silesia, Katowice, POL; 5 Department of Pain Medicine, Paolo Procacci Foundation, Rome, ITA

**Keywords:** ige, clinical trials, immunotherapy, peanut allergy, palforzia

## Abstract

With Palforzia appearing as the first oral immunotherapy for patients with peanut allergy, the present investigation aims to summarize recent clinical trials, the mechanism of dosing, and the real-world usage of this novel therapy. Palforzia offers a new avenue for treating the human allergic response in previous immune modulation refractory patients or patients who have undergone immune environment sensitivity testing, which allows for more specialized treatment. Current studies are focusing on certain age groups that have been shown to be more receptive to treatment. Further, studies are tailoring oral immunotherapy treatment alongside other immune modulators to elicit greater targeted immune tolerance. With an increasing prevalence of patient allergies, many questions remain surrounding the optimization of therapies in reaching therapeutic goals. Overall, Palforzia offers a hopeful treatment for peanut-allergic patients to attenuate their immune response while furthering research in related therapies.

## Introduction and background

Peanut allergy is one the most common and severe forms of food allergy. The prevalence of peanut allergy has also been increasing over the past few decades and is more common in younger populations [1]. Only 20% of the patients will outgrow their allergy to peanuts, so for many, it remains a lifelong burden [2]. With the recent uptick in peanut allergy and the most common lifelong course of the disease, finding therapies for this population is paramount. Palforzia is the first oral immunotherapy (OIT) of its kind to help desensitize patients aged 4-17 years to peanut allergens.

Through a stepwise fashion of gradually introducing more antigens to the immune system of someone who is allergic, Palforzia slowly reduces the immune system's robust anaphylactic response, allowing for greater tolerance to peanuts. While avoidance of peanuts remains the best form of prophylaxis, Palforzia can be valuable in dampening anaphylaxis in accidental exposure. Some of the downsides to Palforzia therapy are that it is a daily medication and anaphylaxis remains a risk factor upon ingestion. For these reasons, studies are also looking into the benefits of other routes of administrating immunotherapy to patients with peanut allergy through sublingual therapy, subcutaneous injections, epicutaneous patches, and recombinant anti-immunoglobulin E (IgE) antibodies. Understanding the body’s immune system allows for using approaches that cause anergy to the body’s response to allergens. Classically, avoidance and post-exposure epinephrine to combat anaphylaxis have been the mainstays for peanut-allergic patients, but with the introduction of Palforzia and continued research into other pathways of subverting the immune system, patients with peanut allergies may soon have a treatment that can build tolerance and possibly cure their allergy.

## Review

Peanut allergy: Mechanism of disease

Understanding the body’s immune system aids in creating effective interventions. The basic physiological response after ingesting any new substance involves the body processing possible pathogenic components and displaying them to the immune system for review. This process begins at the mucosal surface of the gastrointestinal tract, where the food components are taken up by specialized microfold cells (M cells) [3]. Most of the compounds involved in eliciting an immune response are proteins, which allow for the conservation of their original structure as they traverse the digestion tract [4]. After the M cells sample the components of digestion, they transfer the food proteins to dendritic cells for further processing and lymphocyte presentation through surface major histocompatibility complex class II (MHC II) receptors. These dendritic cells migrate to nearby lymph nodes, where they induce cellular changes in naïve T-cells through antigen presentation. In allergic patients, these naïve T-cells are activated into T-helper 2 (Th2) cells, which, through interleukin-4 and interleukin-13 production, begin the cascade of an immune environment that primes B-cells to produce immunoglobulin E (IgE), which are highly sensitive to the antigen. These IgE attach to mast cells and mount an immune response [5].

Upon immune activation, the process of IgE-mediated anaphylaxis begins rapidly. Crosslinking of IgE on mast cells and basophils causes degranulation and subsequent immune mediator production alongside the recruitment of other inflammatory cells. Immediate inflammatory effects include smooth muscle spasms, vasodilation, increased vascular permeability, hypovolemia, myocardial depression, and edema. Anaphylaxis is acutely treated with epinephrine, removal of the allergen, postural changes, and bronchodilator administration [6].

In the past, diagnosing a suspected peanut allergy has classically been done through a double-blind oral food challenge. Recently, however, diagnostic tests have transitioned to a more serological approach. Commonly used modalities include skin prick testing, measuring serum whole peanut IgE levels, measuring serum IgE levels to peanut components, basophil or mast cell activation tests, and histamine release assays. These serological tests have the added benefit of testing patients before an anaphylactic incident or monitoring serum levels as they change in response to immune therapy [7].

Palforzia pharmacodynamics: OIT

Palforzia is a member of the OIT class of drugs. It is the only drug in this class approved for use in the United States [8]. OIT drugs have demonstrated benefits in the treatment of many food-related allergies, such as peanuts, eggs, milk, etc. Treatment with OIT requires daily exposure to the allergen at increasing dosages [9]. The mechanism of action of OIT lies in its constant activation of the immune system. This repetitive activation causes systemic anergy through the desensitization of IgE-mediated mast cells and basophils. While oral immunotherapies are rising in popularity, a competing treatment option is epicutaneous immunotherapy (EPIT). EPIT involves a dosed patch that is placed on the skin of allergic patients. The patients are exposed to increasing levels of allergen, but the benefit of the epicutaneous administration route avoids any systemic reaction that may be caused by other forms of immunotherapy, including OIT [10]. 

Palforzia uses peanut proteins that mimic peanut-induced similar immune responses. In phase 3 trials, patients were exposed to increasing doses starting at 0.5 mg and maximizing at 100 mg [11], a process referred to as crescendo dosing. In the initial meeting with the patient, they are introduced to increasing doses of the allergen until they find the highest dose that does not cause an allergic reaction. The patient then takes the highest established dosage, termed the maintenance dose. The maintenance phase dosage is the top of the crescendo, where the patient’s dosage remains for an extended period, sometimes even lasting years. Clinical trials have indicated that Palforzia had great indications for children but less for adults. Thus, it is indicated by the U.S. Food and Drug Administration (FDA) for children aged 4-17 years. Based on survey responses from prescribing physicians, it is particularly important to closely monitor the conditions in which patients are taking their dosages. Some of these recommendations include the dose after a full meal and avoiding things that may cause reactions, such as nonsteroidal anti-inflammatory drugs [12,13]. The most concerning adverse effect of any therapy that introduces a patient to an allergen is anaphylaxis. In the case of Palforzia, there has been an established association with anaphylaxis as well as moderate gastrointestinal distress [14]. The seriousness of these side effects is an important consideration for physicians when administering this therapy.

Clinical efficacy of Palforzia

With Palforzia being the only drug approved by the FDA for mitigating the immune system's response to peanut allergens, it has been shown that the oral route of immune therapy may be the most effective option. Research is still being done into other routes of administration to see if other pathways may rival the efficacy of OIT. The other routes of administration that are being investigated are subcutaneous immunotherapy (SCIT), sublingual immunotherapy (SLIT), EPIT, and serological approaches through recombinant immunoglobin vaccines that are anti-peanut IgE [15]. The efficacy of Palforzia monotherapy has been explored in two-phase three clinical trials named ARTEMIS (AR101 Treatment Evaluation in Children and Adults - A Randomized, Double-Blind, Placebo-Controlled Study) and PALISADE (Peanut Allergy Oral Immunotherapy Study of AR101 for Desensitization). The ARTEMIS study found that 58.3% of patients tolerated 1,000 mg (three to four peanut kernels) of peanut protein after treatment, with 18.2% of patients experiencing moderate symptoms and another 4.5% of patients experiencing severe symptoms. In the PALISADE study, 50.3% of participants could tolerate the 1,000 mg peanut protein challenge, with moderate symptoms in 25.3% of patients and severe symptoms in 5.1% [16]. Reaching this tolerance threshold to the peanut allergen may increase the patient’s quality of life by lowering anxiety surrounding accidental ingestion when traveling or dining out [17].

Regarding the other pathways of immunotherapy, currently, an EPIT known as Viaskin is undergoing phase three clinical trials with the FDA to find the clinical efficacy of the immunotherapy. Results from the VITESSE study are expected in 2025 [18]. Phase two trials of the Viaskin EPIT patch concluded that after 52 weeks of therapy, patients had modest gains in tolerance to peanut allergens. Younger patients had a greater immunological tolerance response, the majority of the patients had minor patch site reactions, and the immunologic changes noticed in the patients were similar to other routes of immune therapy [19]. An open-label study investigating SLIT found that only 4 mg of peanut protein resulted in clinically significant desensitization that persisted for multiple months following discontinuation of the therapy. The rate of side effects in the SLIT study was low, mostly consisting of a mouth itch. In a preclinical mouse model looking into the efficacy of SCIT, they found that modifying peanut extract and introducing it subcutaneously led to a modification of the animal allergic response, leading to reduced allergenicity to peanut antigens [20,21]. Given the age restrictions and dosing limitations of Palforzia, there is still a large void in the therapeutic options offered to patients with peanut allergy (Table 1) [22].

**Table 1:** Clinical trials relating to peanut oral immunotherapy (OIT) efficacy and safety.

Author (year)	Population and intervention	Results and findings	Conclusions
Vickery et al. [11]	Phase 3, randomized, double-blind, placebo-controlled food challenge; 496 participants aged 4 to 17 years	67.2% of patients receiving treatment were able to tolerate 600 mg of peanut, whereas only 4% of patients taking placebo were able to tolerate the same dose.	Treatment resulted in higher doses of peanuts that could be tolerated in highly allergic participants and lower symptom severity.
Chinthrajah et al. [23]	Phase 2, randomized, double-blind, placebo-controlled trial; 152 patients aged 7 to 55 years with peanut allergy	35% of treated patients were able to tolerate 4,000 mg of peanut at 104 and 117 weeks, while just one placebo participant (4%) was able to do the same.	Treatment with daily 300 mg of oral peanut allergen could desensitize patients up to 4,000 mg of peanut, but discontinuation or reduction of therapy increases the likelihood of becoming clinically reactive to peanut allergen once again.
Monian et al. [24]	Phase 1/2, double-blind, placebo-controlled peanut allergen intervention to elicit and obtain data regarding T-cell function during OIT therapy	Participants were built up to a dose of 4,000 mg of peanut, maintained treatment for 12 weeks and then discontinued treatment and underwent an avoidance phase for 12 weeks to find out the changes in immune system function.	OIT did not reduce the number of reactive Th2 cells but rather led to specific clonal suppression, which may explain why sustained tolerance is difficult to achieve. Some participants who failed to respond to OIT had high baseline Th17 and other T-helper cells, which may be useful in further studies as a predictor for OIT therapy success.
Loke et al. [25]	Multicenter, randomized, phase 2b trial in children aged 1-10 years with known peanut allergy to look into whether probiotics aid in OIT therapy	46% of probiotic plus OIT and 51% of OIT monotherapy patients achieved sustained unresponsiveness to peanut.	Adding a probiotic to the OIT regimen did not improve the efficacy of OIT but may aid in reducing some negative side effects of OIT therapy.
Jones et al. [26]	Randomized, placebo-controlled, double-blind study in children aged 1-3	71% of treated participants vs. 2% of placebo participants reached the targeted end goal, with 21% of treated patients meeting remission requirements. Remission requirements were defined by 26 weeks of no allergen exposure. Then, sensitivity was measured again.	Initiation of OIT therapy in children aged 1-3 years can be associated with higher rates of desensitization and remission from peanut allergy.

## Conclusions

Peanut allergy poses a significant burden on individuals, particularly children, with limited options for long-term management. Palforzia, the first OIT approved by the FDA, offers a promising approach to desensitize patients to peanut allergens. By gradually introducing increasing doses of peanut protein, Palforzia aims to reduce the immune system's hypersensitivity and enhance tolerance to peanuts. However, it is important to note that Palforzia is not without limitations, as it requires daily medication and carries the risk of anaphylaxis. To address these challenges, research is underway to explore alternative routes of immunotherapy, including sublingual therapy, subcutaneous injections, epicutaneous patches, and recombinant anti-IgE antibodies. These approaches aim to modulate the immune response and improve patient outcomes. Diagnostic methods for peanut allergy have also evolved, with serological tests gaining popularity for their accuracy and convenience. While Palforzia has shown efficacy in clinical trials, further investigations are necessary to compare its effectiveness with other administration routes. Moreover, ongoing studies are examining the clinical potential of more specialized immunotherapy based on targeting subsets of patients who may be more receptive to OIT. Ultimately, the development of safe and effective treatments for peanut allergy holds the promise of significantly improving the quality of life for affected individuals, reducing anxiety, and potentially even offering a cure for this lifelong condition.
